# Dietary Sources of Phosphorus among Adults in the United States: Results from NHANES 2001–2014

**DOI:** 10.3390/nu9020095

**Published:** 2017-01-30

**Authors:** Scott T. McClure, Alex R. Chang, Elizabeth Selvin, Casey M. Rebholz, Lawrence J. Appel

**Affiliations:** 1 Welch Center for Prevention, Epidemiology, and Clinical Research, Baltimore, MD 21205, USA; smcclur7@jhu.edu (S.T.M.); achang@geisinger.edu (A.R.C.); eselvin@jhu.edu (E.S.); crebhol1@jhu.edu (C.M.R.); 2 Department of Epidemiology, Johns Hopkins Bloomberg School of Public Health, Baltimore, MD 21205, USA; 3 Division of Nephrology, Johns Hopkins University, Baltimore, MD 21205, USA; 4 Division of Nephrology, Geisinger Health System, Danville, PA 17822, USA; 5 Division of General Internal Medicine, Johns Hopkins University, Baltimore, MD 21205, USA

**Keywords:** NHANES-WWEIA, diet, phosphorus, soda, food groups

## Abstract

Interest in the health effects of dietary phosphorus is burgeoning, yet sources and trends in phosphorus consumption have not been well characterized. We describe trends in and primary sources of dietary phosphorus in a nationally representative sample of 34,741 US adults, 20+ years old (NHANES 2001–2014). Dietary sources of phosphorus were estimated in nine food groups and 26 food categories. Phosphorus consumption was expressed in absolute intake, phosphorus density, and proportion contributed by dietary sources. Between 2001 and 2014, dietary phosphorus intake increased from 1345 to 1399 mg/day (*p*-trend = 0.02), while calorie intake slightly declined (*p*-trend = 0.1). Grains were the largest dietary phosphorus source, followed by meats, and milk products. Soft drinks accounted for just 3.3% of total dietary phosphorus. Phosphorus intake from grains increased 68 mg/day (*p* < 0.001), 25 mg/day from meats (*p* = 0.02), and decreased 75 mg/day (*p* < 0.001) from milk products. Dietary phosphorus intake and the phosphorus density of the diet are increasing. Grains are an important dietary phosphorus source that has increased in total consumption and phosphorus density. Further research is needed to determine if this is due to individuals’ selection of grains or the composition of those available.

## 1. Introduction

Phosphorus is an essential dietary nutrient, and a major component of many structures in the body. Healthy adults have a recommended dietary allowance of at least 700 mg of phosphorus per day, and deficiency is uncommon in the United States [1]. The adverse health effects of high serum phosphorus levels are well documented, and dietary phosphorus restriction is recommended by Kidney Disease | Improving Global Outcomes (KDIGO) guidelines in the treatment of hyperphosphatemia among individuals with chronic kidney disease [2]. Many observational studies have demonstrated an association between serum phosphorus levels and cardiovascular disease, ESRD, and mortality, even within the high-normal range [3,4,5,6,7,8]. 

Extrapolating serum phosphorus outcomes to dietary phosphorus intake is challenging as serum phosphorus is tightly regulated by parathyroid hormone (PTH) and fibroblast growth factor 23 (FGF23) [9]. However, studies have found associations between FGF23 and kidney disease progression, cardiovascular disease, and mortality [10,11,12]. In animal models, high phosphorus intake can cause vascular calcification, renal parenchymal calcification, and proximal tubular injury [13,14,15]. High dietary phosphorus intake has been associated with an increased risk of mortality as well as carotid intima-media thickness and left ventricular mass in healthy populations, although little clinical trial data exists on the effects of phosphorus intake on health outcomes [16,17,18,19,20]. Identifying major dietary sources of phosphorus is an important step to interpreting the literature on the health effects of high dietary phosphorus and helping patients adhere to clinical recommendations to limit dietary intake of phosphorus.

Both the amount of phosphorus in specific foods and the frequency with which those foods are consumed must be taken into account in order to identify important sources of phosphorus in the population. More detailed analysis by sociodemographic factors may be also be informative as phosphorus intake has been shown to differ by race [18]. Little is known about how sources of dietary phosphorus intake vary between sociodemographic groups, and whether sources of phosphorus intake have changed over time. This information will be important for directing further research into dietary phosphorus, as well as for patients and nutritionists.

To identify trends in and important sources of dietary phosphorus in the American diet, we analyzed total dietary phosphorus intake from and phosphorus density of different food sources across seven cycles of the National Health and Nutrition Examination Survey (NHANES)—What We Eat in America (NHANES WWEIA) cycles from 2001 to 2014. We performed analyses using both broad food groups and more narrowly defined food categories and examined differences in consumption of phosphorus by sex, race, and age. 

## 2. Materials and Methods

The National Health and Nutrition Examination Survey (NHANES) is a nationally representative cross-sectional survey of the non-institutionalized, civilian US population. What We Eat in America (WWEIA) is the dietary intake interview portion of NHANES. We analyzed data from the seven NHANES WWEIA survey cycles between 2001 and 2014. We included 34,741 participants (4448 to 5762 per cycle), 20 years of age or older with one complete 24-h dietary interviews. Subgroups were analyzed based on age (20–29, 30–49, 50–69, and 70+), race (Non-Hispanic White, Non-Hispanic Black, and Mexican American), and sex. The protocols for the conduct of NHANES were approved by the National Center for Health Statistics institutional review board (NCHS IRB/ERB), and informed consent was obtained from all participants (NCHS IRB/ERB protocols #98-12, #2005-06, #2011-17).

Trained staff administered 24-h dietary recalls in person, using the United States Department of Agriculture (USDA) Automated Multiple-Pass Method [21]. Nutrient content was then estimated using nutrient profiles from the Food and Nutrient Database for Dietary Studies (FNDDS) [21]. Phosphorus density, the proportion of phosphorus to total caloric intake, was calculated as the ratio of phosphorus (in mg) to energy intake (in kcal).

The USDA defines nine food groups: milk and milk products (milk); meat, poultry, fish and mixtures (meat); eggs; legumes, nuts and seeds (nuts); grain products (grains); fruits; vegetables; fats, oils and salad dressings (fats); and sugar, sweeteners and beverages (beverages). The nine food groups defined by the USDA remained the same across NHANES survey cycles, allowing for an analysis of trends over time. 

For each survey cycle, NHANES WWEIA defined a variety of food categories. Starting in the 2011–2012 survey cycle, NHANES WWEIA defined approximately 150 food categories based on food identification codes in the FNDDS. Unlike the nine USDA food groups which have remained the same over time, these food categories change significantly between survey cycles. For example, between the 2009–2010 cycle and the 2011–2012 cycle, coding changes would have increased reported consumption for burritos and tacos, while decreasing reported consumption of cheese, beans, tortillas, and tomato-based condiments [22]. 

For this reason, only the most recent survey cycle, which included 153 food categories, was used for detailed analysis of dietary sources of phosphorus. We further combined the 153 food categories into 26 food categories based on broad types of foods expected to have similar consumption patterns. For example, milk was separated from other dairy such as cheese; red meat (processed and unprocessed), poultry, and seafood were grouped together. In this paper, we use “food group” to refer to the nine USDA food groups and “food category” to refer to the 26 food categories derived from the WWEIA categories.

Analyses were performed using Stata 14.1 (StataCorp. 2015. Stata Statistical Software: Release 14. College Station, TX, USA: StataCorp LP). Estimated mean total dietary intakes of phosphorus and calcium from foods and beverages were determined using the svy: mean command, individual food files, and survey weights. Age and sex adjustments were made by direct standardization to the 2000 US Census population. Estimated proportion of dietary intakes coming from individual foods were determined using the svy: proportion command, the individual food files, and one-day survey weights. Figures were created using R software, version 3.2.5 (R Project for Statistical Computing). 

The mean estimated dietary intake for commonly consumed nutrients from the 24 h dietary interview are considered unbiased estimates of means of the usual intake distribution for that nutrient [23]. Because means rather than distributions were of interest, the current analyses only examined the first 24 h dietary recalls for participants.

Estimates of the mean phosphorus densities (mg phosphorus per kcal energy intake) and dietary calcium to phosphorus ratios (mg calcium per mg phosphorus intake from foods and beverages) were calculated using the svy: ratio function. Trends across survey cycle for estimated total dietary intakes were calculated from a weighted linear regression modeling survey cycle as an ordinal variable and adjusting for race, age, and sex. Sensitivity analyses looked for trends across survey year for each subgroup adjusting for all other subgroups, as well as modeling survey year as a categorical variable. Adjusted Wald tests were used to test for statistically significant differences between the estimated total dietary intakes across subgroups. Magnitudes of difference were calculated as the percent difference between the comparison group and the reference group.

## 3. Results

Estimated mean dietary phosphorus consumption for the period between 2001 and 2014 was 1373 mg/day (95% CI 1360–1386) with a phosphorus density of 0.633 mg phosphorus per kcal energy intake (95% CI 0.629–0.636). Between 2001 and 2014, there was a statistically significant 4.0% increase in the estimated dietary intake of phosphorus (*p* = 0.0151) and a statistically significant 7.4% increase in phosphorus density (*p* < 0.01), but there was a non-statistically significant decrease in estimated dietary intake of total calories (*p* = 0.139) (Table 1). 

Compared to men, women consumed less phosphorus, but had diets with a similar phosphorus density (Table 2). Non-Hispanic blacks consumed the least amount of phosphorus, and Mexican Americans consumed a similar amount of phosphorus to non-Hispanic whites. Phosphorus consumption tended to decrease with age, while phosphorus density tended to increase; the reduction of phosphorus with age likely results from a reduction in total calorie intake (Table S1). Direct age and sex standardization to the 2000 US census population yielded similar results.

Of the nine USDA food groups, grains, meat, and milk contributed the most phosphorus to the diet across all subgroups, accounting for 76% of dietary phosphorus overall (Table 3). Men and women tended to consume foods with similar phosphorus densities (Table 4). Compared to non-Hispanic whites, non-Hispanic blacks consumed meat, milk, and beverages with lower phosphorus densities. Mexican Americans consumed grains with a higher phosphorus density and beverages with a lower phosphorus density compared with non-Hispanic Whites. Compared to 20–29 year olds, those 70 and older consumed meat and beverages with a higher phosphorus density. A sensitivity analysis restricted to the 2013–2014 survey cycle found similar trends (Tables S2 and S3). 

Table 5 and Table S4 describe the amount and proportion of phosphorus contributed by 26 food categories in the 2013–2014 survey cycle. The top five food categories—meat/poultry/seafood, milk, bread, non-dairy snacks and sweets, and other dairy—contributed a combined 43.1% of dietary phosphorus overall. Soft drinks (defined as all non-alcoholic beverages excluding coffee, tea, and 100% juice) contributed just 3.3% of dietary phosphorus overall compared to 7.0% from non-dairy snacks and sweets and 7.6% from milk. The largest subgroup differences were seen between races. Compared to non-Hispanic whites, non-Hispanic blacks consumed a greater proportion of their dietary phosphorus from Meat/poultry/seafood and less from milk. Mexican Americans consumed a greater proportion of dietary phosphorus from breads and Mexican mixed dishes.

**Figure 1:**
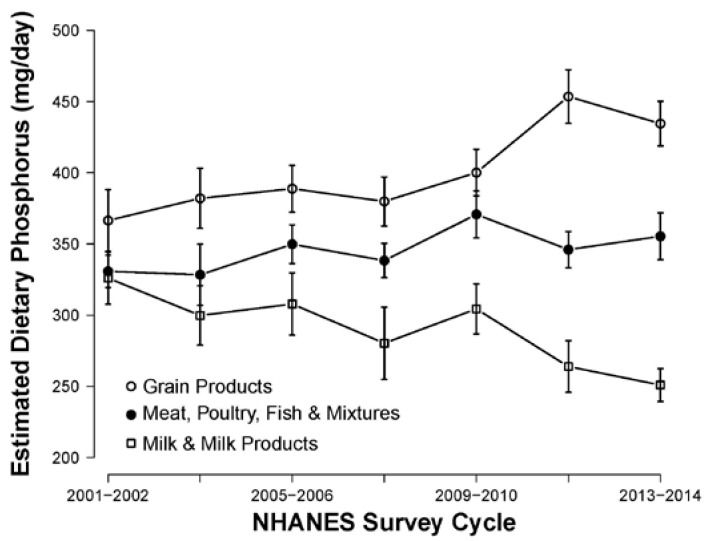
Estimated total dietary phosphorus contributed by the top 3 USDA food groups. Error bars represent a 95% confidence interval.

**Figure 2:**
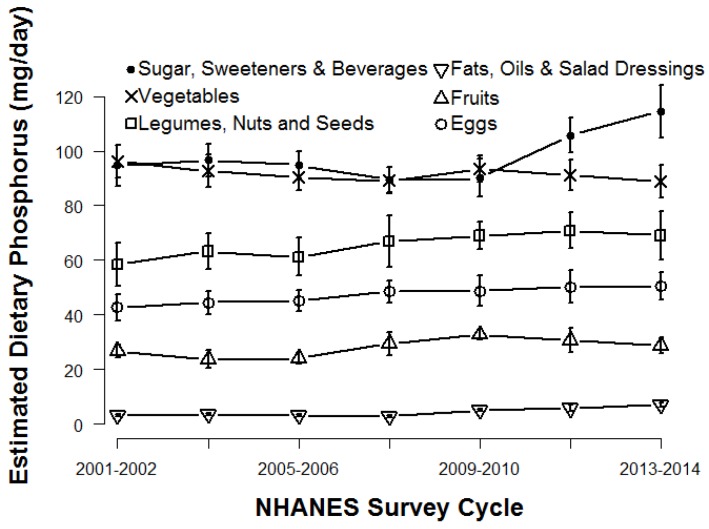
Estimated total dietary phosphorus contributed by the bottom 6 USDA food groups. Error bars represent a 95% confidence interval.

Figure 1 and Figure 2 show the amount of dietary phosphorus contributed by the nine USDA food groups across all seven NHANES survey cycles from 2001 to 2014. Milk, meat, and grains contributed the most dietary phosphorus in all cycles. There was an increasing trend in the contribution from grains and meat and a declining trend from milk. Between 2001 and 2010, dietary phosphorus contributed by beverages remained stable, but showed an increasing trend after 2010. Grains contributed the most calories across all seven NHANES survey cycles from 2001 to 2014, followed by meat and beverages (Figures S1 and S2).

The estimated mean dietary calcium to phosphorus ratio (mg calcium per mg phosphorus intake from foods and beverages) for the period between 2001 and 2014 was 0.689 mg calcium per mg phosphorus intake from foods and beverages (95% CI 0.685–0.694). Between 2001 and 2014, there was a statistically significant 6.4% increase in the dietary calcium to phosphorus ratio (*p* < 0.01) (Table S5). 

Compared to men, women had diets with a higher dietary calcium to phosphorus ratios (Table S6). Non-Hispanic blacks and Mexican Americans consumed diets with a lower dietary calcium to phosphorus ratios compared with non-Hispanic whites. Direct age and sex standardization to the 2000 US census population yielded similar results.

Of the nine USDA food groups, milk, grain, meat, and beverages contributed the most calcium to the diet across all subgroups, accounting for 78% of dietary calcium intake from foods and beverages overall (Table S7). Men and women tended to consume foods with similar dietary calcium to phosphorus densities (Table S8). Compared to non-Hispanic whites, non-Hispanic blacks consumed beverages with higher dietary calcium to phosphorus ratios (Table S8).

## 4. Discussion

In this analysis of a nationally representative sample with dietary intake data from 2001 to 2014, we documented an increase both in total phosphorus consumption and the phosphorus density of the diet between 2001 and 2014 in U.S. adults. Our results highlight the importance of grain products (specifically, breads and non-dairy snacks and sweets) as a source of dietary phosphorus. We showed that soft drinks are not currently a major source of dietary phosphorus, accounting for only 3.3% of total phosphorus consumption. However, the contribution from beverages is increasing. We also demonstrated that sources of phosphorus intake varied substantially by race/ethnicity. 

Dietary phosphorus education for individuals with hyperphosphatemia often emphasizes the phosphorus content of specific foods, as well as identifying and avoiding foods with phosphorus-containing food ingredients [24,25]. However, there is variability in the contribution of phosphorus depending on the type and quantity of phosphorus-containing ingredients. For instance, phosphoric acid is used as a flavoring in dark sodas at approximately 0.05% by weight [26], while sodium acid pyrophosphate is used in baked goods at levels well over 1% [27]. Prior studies and commentaries have emphasized the importance of soda and other soft drinks as important dietary sources of phosphorus [16,28,29,30,31,32,33]. Our study found a relatively minor contribution of soda and other soft drinks to overall phosphorus intake. Surprisingly, though grains tend to have a relatively low phosphorus density overall, their high frequency of consumption makes them a predominant source in the U.S. adult diet. 

There is a wide variation in phosphorus content of grain products. Whole grain containing foods have a higher phosphorus density than similar refined grain containing foods due to the phosphorus content of grain components removed during refining. For example, whole grain wheat flour has a phosphorus density 3.5 times higher than white, all-purpose, enriched, bleached wheat flour [34]. Also, baked goods (including chemically leavened breads such as muffins and biscuits and non-dairy snacks and sweets such as cookies and crackers) have major differences in phosphorus content depending on the leavening ingredients used. One study found a pooled sample of muffins contained 70% more phosphorus per 100 g than a pooled sample of cookies, and 367% more digestible phosphorus per 100 g [33]. The muffins in that study contained a phosphorus leavener (sodium polyphosphate), while the cookies did not [33]. Complicating this further is the discordance between phosphorus bioavailability seen in feeding studies of urine excretion and phosphorus bioavailability estimated by phosphorus digestibility models [35]. Carefully controlled balance studies are needed to better understand bioavailability of phosphorus from various sources. 

A major limitation of this study is the potential misreporting of phosphorus content by nutrient databases. Indeed, the underestimation of phosphorus content of beverages and meats by nutrition databases has been previously reported [29,31]. Dependence on self-reported dietary consumption is a limitation of all studies using 24-h dietary recall. However, the USDA Automated Multiple Pass method, used in NHANES since 2002, has been shown to improve standardization across interviewers and enhance participant recall as compared to other methods [36,37,38]. We analyzed trends using linear regression, though the data may not be linear. 

Strengths of this study include the large sample size and nationally representative data source, including the most recent national data available. This allowed for evaluations of trends over time by age, sex, and race. The use of multiple food categorizations allowed for analyses of general trends over time and a detailed examination of the most recent data. This study found changes in dietary sources of phosphorus from 2001 to 2014 that are in line with other changes seen in the US diet. These include the reduced consumption of soft drinks and milk, and increased consumption of whole grains [39].

Our findings have implications for clinical practice and future research. For those with hyperphosphatemia, our results highlight the importance of education on the phosphorus content of grain products, especially the differences between baked goods leavened with phosphorus and those leavened without phosphorus. Additional research into the possible sources of variation between grain products is of particular importance. Engaging with the food industry will be critical in improving the accuracy of phosphorus content in nutritional databases, especially for those foods with phosphorus-containing ingredients. 

In conclusion, dietary phosphorus intake and phosphorus density of the U.S. diet is increasing. Grain products are an important dietary source of phosphorus that has increased over time. This increase is not only due to higher overall consumption, but also consumption of grain products with higher phosphorus density. Further research is needed to determine if these changes are due to individuals’ selection of foods, the composition of available foods, or both. 

## Figures and Tables

**Table 1:** Mean daily phosphorus and total calorie intake and phosphorus density (proportion of phosphorus of total calories) for U.S. adults 20 years of age or older and according to NHANES WWEIA survey cycle.

NHANES WWEIA Survey Cycle	*N* (Unweighted)	Mean Phosphorus (95% CI) (mg/day)	Mean Calories (95% CI) (kcal/day)	Phosphorus Density (95% CI) (mg/kcal)
Overall	34,741	1373 (1360–1386)	2182 (2166–2198)	0.633 (0.629–0.636)
2001–2002	4744	1345 (1309–1382)	2208 (2159–2257)	0.609 (0.601–0.618)
2003–2004	4448	1334 (1300–1368)	2216 (2183–2249)	0.602 (0.589–0.615)
2005–2006	4520	1366 (1323–1409)	2195 (2129–2260)	0.622 (0.615–0.629)
2007–2008	5419	1324 (1278–1371)	2115 (2056–2174)	0.626 (0.616–0.636)
2009–2010	5762	1414 (1387–1441)	2132 (2093–2171)	0.663 (0.654–0.673)
2011–2012	4801	1418 (1392–1444)	2191 (2160–2222)	0.647 (0.639–0.655)
2013–2014	5047	1399 (1376–1423)	2141 (2104–2178)	0.654 (0.643–0.664)
Change		4.01%	−3.03%	7.39%
*p*-Value for Trend *		0.0151	0.139	<0.01

* *p*-Values are calculated from a survey weighted linear regression modeling survey cycle ordinally adjusted for race, age, and sex.

**Table 2:** Crude and age-adjusted estimated means (95% CIs) for total dietary phosphorus and phosphorus density for adults 20 years of age or older, NHANES WWEIA 2001–2014.

Population Subgroup	*N* (Unweighted)	Mean Total Dietary Phosphorus (mg/day)		Mean Phosphorus Density (mg/kcal)	
		Crude	Adjusted	Crude	Adjusted
**All**	34,741	1373 (1360–1386)	1379 (1366–1391)	0.633 (0.629–0.636)	0.635 (0.631–0.638)
**Male (REF)**	16,806	1603 (1586–1621)	1615 (1597–1633)	0.627 (0.623–0.631)	0.628 (0.624–0.632)
**Female**	17,935	1159 (1146–1171) **	1159 (1147–1171) **	0.640 (0.635–0.645) **	0.641 (0.636–0.645) **
**NH White (REF)**	16,569	1403 (1387–1419)	1419 (1404–1435)	0.642 (0.637–0.647)	0.643 (0.638–0.647)
**NH Black**	7213	1211 (1186–1237) **	1214 (1190–1237) **	0.562 (0.556–0.568) **	0.567 (0.562–0.573) **
**Mexican American**	5914	1467 (1434–1500) ^n.s.^	1410 (1383–1438) ^n.s.^	0.656 (0.647–0.665) *	0.666 (0.658–0.674) **
**Other**	5045	1273 (1248–1298) **	1263 (1241–1286) **	0.626 (0.619–0.633) **	0.632 (0.626–0.639) *
**20–29 (REF)**	6229	1452 (1423–1480)	1456 (1428–1485)	0.609 (0.603–0.616)	0.609 (0.602–0.615)
**30–49**	11,849	1456 (1437–1476) ^n.s.^	1460 (1441–1478) ^n.s.^	0.628 (0.623–0.633) **	0.629 (0.624–0.634) **
**50–69**	10,587	1312 (1292–1332) **	1312 (1292–1331) **	0.646 (0.639–0.652) **	0.648 (0.642–0.654) **
**70+**	6076	1132 (1115–1149) **	1124 (1108–1140) **	0.665 (0.659–0.671) **	0.665 (0.659–0.671) **

* *p* < 0.05, ** *p* < 0.01, ^n.s.^ *p* ≥ 0.05, bold = more than 5% different than the reference group. Adjusted values are age and sex standardized to the 2000 Standard US Census population. NH = Non-Hispanic.

**Table 3:** Estimated total dietary phosphorus (mg/day) by USDA food group in NHANES WWEIA 2001–2014 in adults 20 years of age or older.

Food Group	All	Male	Female	NH White	NH Black	Mexican American	Other	20–29	30–49	50–69	70+
Milk & Milk Products	289	320	261	324	175	245	224	301	296	278	280
	(21.0%)										
Meat, Poultry, Fish & Mixtures	346	428	270	337	401	337	355	368	372	332	263
	(25.2%)										
Eggs	47	58	38	45	52	69	44	45	49	49	39
	(3.4%)										
Legumes, Nuts and Seeds	66	76	56	66	50	82	69	57	69	71	56
	(4.8%)										
Grain Products	402	466	343	399	341	530	388	455	431	365	316
	(29.3%)										
Fruits	28	30	26	27	28	32	32	26	26	29	34
	(2.0%)										
Vegetables	92	100	84	97	85	71	81	87	92	95	88
	(6.7%)										
Fats, Oils & Salad Dressings	4	5	4	5	4	3	3	4	5	5	4
	(0.3%)										
Sugar, Sweeteners & Beverages	98	121	77	105	76	96	77	108	116	89	52
	(7.1%)										
**All Sources**	1373	1603	1159	1403	1211	1467	1273	1452	1456	1312	1132

NH = Non-Hispanic.

**Table 4:** Estimated dietary phosphorus density (mg phosphorus/kcal) by USDA food group in NHANES WWEIA 2001–2014 in adults 20 years of age or older.

Food Group	All	Male	Female	NH White	NH Black	Mexican American	Other	20–29	30–49	50–69	70+
Milk & Milk Products	1.308	1.322	1.292	1.322	1.188	1.310	1.283	1.344	1.296	1.292	1.330
Meat, Poultry, Fish & Mixtures	0.854	0.848	0.863	0.861	0.788	0.854	0.894	0.828	0.849	0.872	0.881
Eggs	1.048	1.048	1.048	1.055	1.054	1.005	1.045	1.045	1.046	1.046	1.068
Legumes, Nuts and Seeds	0.812	0.804	0.823	0.803	0.837	0.815	0.850	0.863	0.834	0.782	0.757
Grain Products	0.532	0.536	0.528	0.529	0.502	0.616	0.512	0.535	0.535	0.528	0.530
Fruits	0.304	0.305	0.303	0.305	0.300	0.303	0.301	0.303	0.305	0.304	0.303
Vegetables	0.563	0.537	0.595	0.575	0.498	0.563	0.555	0.533	0.550	0.583	0.609
Fats, Oils & Salad Dressings	0.076	0.072	0.080	0.072	0.091	0.098	0.088	0.090	0.080	0.069	0.062
Sugar, Sweeteners & Beverages	0.281	0.272	0.295	0.301	0.190	0.270	0.267	0.236	0.285	0.313	0.304
**All Sources**	**0.633**	**0.627**	**0.640**	**0.642**	**0.562**	**0.656**	**0.626**	**0.609**	**0.628**	**0.646**	**0.665**

NH = Non-Hispanic.

**Table 5:** Estimated total dietary phosphorus (mg/day) contributed by types of food for adults aged 20 and older from NHANES-WWEIA 2013–2014.

Food Category	All	Male	Female	NH White	NH Black	Mexican American	Other	20–29	30–49	50–69	70+
Beverages—Alcoholic	28	43	13	30	29	29	17	34	32	27	10
Beverages—All Other	46	50	42	49	36	42	44	56	46	45	34
Beverages—Coffee/Tea	23	23	23	26	14	17	21	19	29	22	16
Dairy—Milk	110	135	86	125	61	93	90	130	99	100	135
Dairy—Other	111	118	104	127	77	84	76	110	108	118	97
Fruits and Vegetables—Excluding Potatoes	49	48	51	51	42	38	55	39	49	53	59
Fruits and Vegetables—White Potatoes	31	37	25	33	34	25	22	32	32	30	29
Grains—Breads	101	116	86	94	92	163	99	86	107	102	102
Grains—Cooked Grains/Cereals	27	28	27	22	30	20	55	19	30	27	36
Grains—RTE Cereals	28	33	24	34	16	18	16	22	20	32	50
Mixed Dishes—Asian	21	23	19	18	13	18	42	27	23	20	10
Mixed Dishes—Grain-Based	47	56	40	48	58	34	47	51	44	52	38
Mixed Dishes—Meat, Poultry, Seafood	55	62	48	58	54	42	46	40	51	65	59
Mixed Dishes—Mexican	70	84	56	57	27	228	62	101	88	49	24
Mixed Dishes—Pizza	63	75	51	64	61	55	65	120	73	37	16
Mixed Dishes—Sandwiches	55	64	47	50	84	83	39	73	65	44	32
Mixed Dishes—Soups	22	24	20	19	11	37	32	16	23	24	23
Other—All Other	16	24	10	14	5	50	14	34	18	9	5
Other—Fats, Oils, Condiments, Sugars	19	20	18	19	17	18	18	20	22	18	13
Protein Foods—Cured Meat	55	70	41	63	50	35	36	54	59	54	49
Protein Foods—Eggs	45	54	36	42	45	63	47	48	44	45	43
Protein Foods—Meat/Poultry/Seafood	184	225	145	169	257	158	212	207	200	170	136
Protein Foods—Plant Based	64	73	56	62	51	85	72	49	69	70	56
Snacks and Sweets—Excluding Frozen Dairy	98	103	93	102	108	81	80	102	99	100	83
Snacks and Sweets—Frozen Dairy	20	23	17	23	16	10	14	13	21	19	30

Dark grey shading indicates highest single contributor, light grey shading indicates one of the top 5 contributors. NH = Non-Hispanic.

## Supplementary Materials

The following are available online at http://www.mdpi.com/2072-6643/9/2/95/s1, Table S1: Crude and age/sex adjusted estimated values with 95% CI’s for total dietary calories for adults 20 years of age or older for NHANES WWEIA 2001–2014; Table S2: Estimated Total Dietary Phosphorus (mg/day) by USDA Food Groups in NHANES WWEIA 2013–2014 in adults 20 years of age or older; Table S3: Estimated Phosphorus Density Ratio (mg·P/kcal) by USDA Food Groups in NHANES WWEIA 2013–2014 in adults 20 years of age or older; Table S4: Proportion of total dietary phosphorus contributed by types of food for adults aged 20 and older from NHANES-WWEIA 2013–2014; Table S5: Mean daily dietary calcium intake from foods and beverages and dietary calcium to phosphorus ratio for U.S. adults 20 years of age or older and according to NHANES WWEIA survey cycle; Table S6: Crude and age adjusted estimated means (95% CIs) for total dietary calcium intake from foods and beverages and dietary calcium to phosphorus ratio for adults 20 years of age or older, NHANES WWEIA 2001–2014; Table S7: Estimated Total Dietary Calcium Intake from Foods and Beverages (mg/day) by USDA Food Groups in NHANES WWEIA 2001–2014 in adults 20 years of age or older; Table S8: Estimated Dietary Calcium to Phosphorus ratio (mg calcium per mg phosphorus intake from foods and beverages) by USDA Food Groups in NHANES WWEIA 2001–2014 in adults 20 years of age or older; Figure S1: Estimated Total Dietary Energy Intake contributed by the top 3 USDA food groups; Figure S2: Estimated Total Dietary Energy Intake contributed by the bottom 6 USDA food groups.
