# Supplementary material for: Dietary Sources of Phosphorus among Adults in the United States: Results from NHANES 2001–2014
Source: Nutrients. 2017 Jan 30;9(2):95. doi: 10.3390/nu9020095 (PMC5331526; doi:10.3390/nu9020095)
Supplement: Supplementary file 1 [file nutrients-09-00095-s001.pdf]

Dietary Sources of Phosphorus among Adults in the United States: Results from NHANES 2001–2014

Scott T. McClure, Alex R. Chang, Elizabeth Selvin, Casey M. Rebholz and Lawrence J. Appel

Table S1. Crude and age/sex adjusted estimated values with 95% CI's for total dietary calories for adults 20 years of age or older for NHANES WWEIA 2001–2014.

Population Subgroup	Calories (kcal/day)	
	Crude	Adjusted
**All**	2182 (2166–2198)	2170 (2153–2187)
**Male (REF)**	2578 (2553–2604)	2557 (2533–2582)
**Female**	**1813 (1799–1827) ****	**1811 (1796–1826) ****
**NH White (REF)**	2218 (2199–2237)	2186 (2165–2207)
**NH Black**	2153 (2120–2187) **	2155 (2115–2195) **
**Mexican American**	2128 (2093–2162) **	2237 (2198–2276) **
**Other**	**2008 (1975–2040) ****	**2033 (1997–2069) ****
**20–29 (REF)**	2390 (2349–2431)	2382 (2341–2423)
**30–49**	2325 (2300–2349) **	2319 (2293–2345) **
**50–69**	**2032 (2005–2058) ****	**2032 (2004–2060) ****
**70+**	**1690 (1668–1712) ****	**1703 (1679–1726) ****

** $p < 0.01$, n.s. $p \geq 0.05$, **bold** = more than 5% different than the reference group; Adjusted values are age and sex standardized to the 2000 US Census population. NH = Non-Hispanic.

Table S2. Estimated Total Dietary Phosphorus (mg/day) by USDA Food Groups in NHANES WWEIA 2013–2014 in adults 20 years of age or older.

Food Group	All	Male	Female	NH White	NH Black	Mexican American	Other	20–29	30–49	50–69	70+
Milk & Milk Products	251	287	217	286	159	196	194	273	239	244	271
Meat, Poultry, Fish & Mixtures	355	427	288	345	441	309	365	377	383	340	284
Eggs	50	59	42	47	57	66	50	55	49	50	48
Legumes, Nuts and Seeds	69	79	60	66	53	88	83	53	75	75	60
Grain Products	435	497	375	417	379	633	431	499	466	399	340
Fruits	29	30	27	27	28	39	31	25	28	28	38
Vegetables	89	96	82	93	90	71	82	86	88	89	94
Fats, Oils & Salad Dressings	7	7	7	7	10	7	6	8	7	6	6
Sugar, Sweeteners & Beverages	115	142	89	121	90	133	90	139	124	109	64
All Source	1399	1625	1187	1410	1306	1542	1332	1515	1461	1340	1205

NH = Non-Hispanic.

Table S3. Estimated Phosphorus Density Ratio (mg·P/kcal) by USDA Food Groups in NHANES WWEIA 2013–2014 in adults 20 years of age or older.

Food Group	All	Male	Female	NH White	NH Black	Mexican American	Other	20–29	30–49	50–69	70+
Milk & Milk Products	1.217	1.235	1.195	1.227	1.180	1.256	1.15	1.281	1.165	1.223	1.243
Meat, Poultry, Fish & Mixtures	0.924	0.915	0.936	0.941	0.854	0.901	0.938	0.901	0.937	0.924	0.923
Eggs	1.023	1.031	1.012	1.030	1.012	0.985	1.036	1.006	1.011	1.032	1.066
Legumes, Nuts and Seeds	0.790	0.793	0.787	0.778	0.780	0.848	0.808	0.829	0.820	0.768	0.724
Grain Products	0.553	0.560	0.544	0.543	0.513	0.651	0.551	0.573	0.564	0.534	0.534
Fruits	0.340	0.349	0.330	0.342	0.316	0.383	0.319	0.333	0.352	0.325	0.354
Vegetables	0.568	0.544	0.596	0.578	0.489	0.560	0.604	0.545	0.560	0.571	0.619
Fats, Oils & Salad Dressings	0.132	0.124	0.140	0.116	0.188	0.180	0.147	0.162	0.149	0.119	0.086
Sugar, Sweeteners & Beverages	0.343	0.343	0.343	0.365	0.228	0.388	0.315	0.33	0.329	0.364	0.371
All Sources	0.654	0.656	0.65	0.662	0.586	0.684	0.65	0.651	0.651	0.652	0.674

NH = Non-Hispanic.

Table S4. Proportion of total dietary phosphorus contributed by types of food for adults aged 20 and older from NHANES-WWEIA 2013–2014.

Food Category	All	Male	Female	NH White	NH Black	Mexican American	Other	20–29	30–49	50–69	70+
Beverages—100% Juice	0.88%	0.89%	0.87%	0.77%	1.27%	1.20%	0.91%	0.82%	0.80%	0.74%	1.72%
Beverages—Alcoholic	1.99%	2.67%	1.10%	2.11%	2.19%	1.88%	1.29%	2.22%	2.19%	1.98%	0.79%
Beverages—All Other	3.28%	3.10%	3.53%	3.44%	2.79%	2.71%	3.33%	3.70%	3.14%	3.33%	2.79%
Beverages—Coffee/Tea	1.65%	1.42%	1.94%	1.84%	1.08%	1.08%	1.57%	1.22%	1.98%	1.65%	1.34%
Dairy—Milks	7.84%	8.31%	7.23%	8.84%	4.68%	6.03%	6.73%	8.61%	6.78%	7.47%	11.23%
Dairy—Other	7.90%	7.26%	8.74%	9.04%	5.92%	5.41%	5.69%	7.29%	7.38%	8.81%	8.09%
Fruits and Vegetables—Excluding Potatoes	3.54%	2.93%	4.31%	3.64%	3.21%	2.46%	4.11%	2.60%	3.33%	3.93%	4.90%
Fruits and Vegetables—White Potatoes	2.22%	2.28%	2.14%	2.36%	2.64%	1.61%	1.63%	2.14%	2.21%	2.22%	2.39%
Grains—Breads	7.19%	7.17%	7.21%	6.64%	7.02%	10.56%	7.47%	5.66%	7.30%	7.61%	8.49%
Grains—Cooked Grains/Cereals	1.96%	1.73%	2.27%	1.59%	2.26%	1.30%	4.12%	1.22%	2.06%	2.01%	2.97%
Grains—RTE Cereals	2.00%	2.01%	1.99%	2.41%	1.24%	1.15%	1.19%	1.44%	1.40%	2.37%	4.11%
Mixed Dishes—Asian	1.49%	1.39%	1.63%	1.29%	1.02%	1.16%	3.17%	1.77%	1.56%	1.46%	0.83%
Mixed Dishes—Grain-Based	3.39%	3.42%	3.35%	3.38%	4.41%	2.23%	3.49%	3.39%	2.98%	3.91%	3.16%
Mixed Dishes—Meat, Poultry, Seafood	3.90%	3.80%	4.03%	4.13%	4.12%	2.69%	3.46%	2.64%	3.47%	4.87%	4.88%
Mixed Dishes—Mexican	4.99%	5.19%	4.73%	4.04%	2.08%	14.80%	4.62%	6.69%	6.05%	3.68%	1.96%
Mixed Dishes—Pizza	4.49%	4.64%	4.29%	4.52%	4.67%	3.55%	4.89%	7.89%	4.99%	2.76%	1.33%

Mixed Dishes—Sandwiches	3.96%	3.95%	3.98%	3.55%	6.44%	5.38%	2.95%	4.85%	4.45%	3.27%	2.69%
Mixed Dishes—Soups	1.55%	1.47%	1.65%	1.37%	0.82%	2.40%	2.41%	1.04%	1.54%	1.77%	1.89%
Other—All Other	1.17%	1.46%	0.81%	1.01%	0.40%	3.21%	1.03%	2.26%	1.22%	0.68%	0.39%
Other—Fats, Oils, Condiments, Sugars	1.35%	1.23%	1.51%	1.38%	1.32%	1.19%	1.37%	1.29%	1.48%	1.33%	1.07%
Protein Foods—Cured Meat	3.94%	4.32%	3.44%	4.46%	3.86%	2.25%	2.69%	3.58%	4.02%	4.03%	4.09%
Protein Foods—Eggs	3.21%	3.32%	3.07%	2.97%	3.47%	4.07%	3.56%	3.16%	3.05%	3.32%	3.55%
Protein Foods—Meat/Poultry/Seafood	13.13%	13.85%	12.20%	11.98%	19.65%	10.23%	15.90%	13.67%	13.72%	12.72%	11.27%
Protein Foods—Plant Based	4.57%	4.48%	4.68%	4.36%	3.92%	5.50%	5.38%	3.24%	4.70%	5.24%	4.63%
Snacks and Sweets—Excluding Frozen Dairy	6.99%	6.32%	7.87%	7.24%	8.30%	5.27%	6.01%	6.76%	6.75%	7.44%	6.90%
Snacks and Sweets—Frozen Dairy	1.42%	1.39%	1.46%	1.65%	1.21%	0.65%	1.03%	0.85%	1.45%	1.40%	2.53%

Dark grey shading indicates highest single contributor, light grey shading indicates one of the top 5 contributors. NH = Non-Hispanic.

Table S5. Mean daily dietary calcium intake from foods and beverages and dietary calcium to phosphorus ratio for U.S. adults 20 years of age or older and according to NHANES WWEIA survey cycle.

NHANES WWEIA Survey Cycle	N (Unweighted)	Mean Dietary Calcium (95% CI) (mg/day)	Dietary Calcium to Phosphorus Ratio (95%CI) (mg Ca/mg P)
Overall	34,741	946 (934–959)	0.689 (0.685–0.694)
2001–2002	4744	870 (834–906)	0.652 (0.640–0.663)
2003–2004	4448	885 (849–921)	0.664 (0.648–0.680)
2005–2006	4520	966 (932–1001)	0.711 (0.698–0.724)
2007–2008	5419	933 (887–979)	0.707 (0.691–0.722)
2009–2010	5762	1019 (1000–1038)	0.724 (0.716–0.731)
2011–2012	4801	997 (967–1028)	0.703 (0.690–0.715)
2013–2014	5047	970 (948–993)	0.694 (0.685–0.703)
Change		11.49%	6.44%
p-Value for Trend *		<0.01	<0.01

* *p*-Values are calculated from a survey weighted linear regression modeling survey cycle ordinally adjusted for race, age, and sex.

Table S6. Crude and age adjusted estimated means (95% CIs) for total dietary calcium intake from foods and beverages and dietary calcium to phosphorus ratio for adults 20 years of age or older, NHANES WWEIA 2001–2014.

Population Subgroup	Unweighted N	Mean Total Dietary Calcium (mg/day)		Mean Dietary Calcium to Phosphorus Ratio (mg Ca/mg P)	
		Crude	Adjusted	Crude	Adjusted
**All**	34,741	946 (934–959)	951 (938–963)	0.689 (0.685–0.694)	0.694 (0.690–0.699)
**Male (REF)**	16,806	1066 (1050–1083)	1074 (1057–1092)	0.665 (0.659–0.671)	0.665 (0.659–0.671)
**Female**	17,935	**835 (822–848) ****	**836 (823–849) ****	**0.721 (0.714–0.727) ****	**0.722 (0.716–0.728) ****
**NH White (REF)**	16,569	989 (973–1005)	1003 (987–1019)	0.705 (0.699–0.711)	0.711 (0.705–0.717)
**NH Black**	7213	**776 (756–795) ****	**775 (757–793) ****	**0.640 (0.631–0.650) ****	**0.642 (0.633–0.652) ****
**Mexican American**	5914	967 (940–995) **	934 (908–960) **	**0.660 (0.647–0.672) ****	**0.670 (0.657–0.682) ****
**Other**	5045	**834 (810–859) ****	**828 (805–851) ****	**0.655 (0.644–0.667) ****	**0.660 (0.648–0.671) ****
**20–29 (REF)**	6229	1035 (1007–1062)	1038 (1010–1065)	0.713 (0.701–0.724)	0.716 (0.705–0.727)
**30–49**	11,849	991 (971–1010) **	992 (973–1011) **	0.680 (0.673–0.687) **	0.685 (0.678–0.692) **
**50–69**	10,587	**890 (871–909) ****	**890 (870–909) ****	0.678 (0.670–0.686) **	0.682 (0.674–0.690) **
**70+**	6076	**807 (792–0823) ****	**804 (789–819) ****	0.713 (0.705–0.721) $^{n.s.}$	0.720 (0.711–0.728) $^{n.s.}$

** $p < 0.01$, $^{n.s.}$ $p \geq 0.05$, bold = more than 5% different than the reference group. Adjusted values are age and sex standardized to the 2000 Standard US Census population. NH = Non-Hispanic.

Table S7. Estimated Total Dietary Calcium Intake from Foods and Beverages (mg/day) by USDA Food Groups in NHANES WWEIA 2001–2014 in adults 20 years of age or older.

Food Group	All	Male	Female	NH White	NH Black	Mexican American	Other	20–29	30–49	50–69	70+
Milk & Milk Products	364 (38.5%)	406	325	405	223	314	288	385	373	347	348
Meat, Poultry, Fish & Mixtures	73 (7.7%)	89	59	72	87	67	71	86	79	67	51
Eggs	21 (2.2%)	25	16	20	23	28	18	21	22	21	15
Legumes, Nuts and Seeds	21 (2.2%)	24	18	21	15	26	24	21	22	21	19
Grain Products	270 (28.5%)	316	228	276	228	328	231	321	288	239	212
Fruits	38 (4.0%)	40	36	34	45	47	45	40	35	37	43
Vegetables	53 (5.6%)	54	52	56	51	40	48	46	52	58	55
Fats, Oils & Salad Dressings	3 (0.3%)	3	3	3	2	2	2	2	3	3	2
Sugar, Sweeteners & Beverages	103 (10.9%)	110	97	102	101	115	107	112	117	97	62
**All Sources**	**946**	**1066**	**835**	**989**	**776**	**967**	**834**	**1035**	**991**	**890**	**807**

NH = Non-Hispanic.

Table S8. Estimated Dietary Calcium to Phosphorus ratio (mg calcium per mg phosphorus intake from foods and beverages) by USDA Food Groups in NHANES WWEIA 2001–2014 in adults 20 years of age or older.

Food Group	All	Male	Female	NH White	NH Black	Mexican American	Other	20–29	30–49	50–69	70+
Milk & Milk Products	1.259	1.268	1.248	1.252	1.279	1.282	1.287	1.282	1.259	1.248	1.242
Meat, Poultry, Fish & Mixtures	0.212	0.208	0.218	0.214	0.217	0.199	0.201	0.234	0.213	0.201	0.195
Eggs	0.435	0.433	0.438	0.443	0.452	0.400	0.402	0.464	0.443	0.422	0.392
Legumes, Nuts and Seeds	0.322	0.316	0.330	0.321	0.291	0.322	0.356	0.373	0.314	0.302	0.342
Grain Products	0.672	0.678	0.665	0.692	0.669	0.619	0.597	0.704	0.668	0.655	0.669
Fruits	1.338	1.331	1.345	1.262	1.624	1.470	1.389	1.532	1.343	1.265	1.247
Vegetables	0.579	0.542	0.621	0.576	0.601	0.562	0.596	0.532	0.563	0.611	0.622
Fats, Oils & Salad Dressings	0.643	0.648	0.637	0.680	0.498	0.442	0.665	0.552	0.654	0.676	0.669
Sugar, Sweeteners & Beverages	1.051	0.905	1.265	0.966	1.331	1.191	1.380	1.042	1.007	1.096	1.205
**All Sources**	**0.689**	**0.665**	**0.721**	**0.705**	**0.640**	**0.660**	**0.655**	**0.713**	**0.680**	**0.678**	**0.713**

NH = Non-Hispanic.

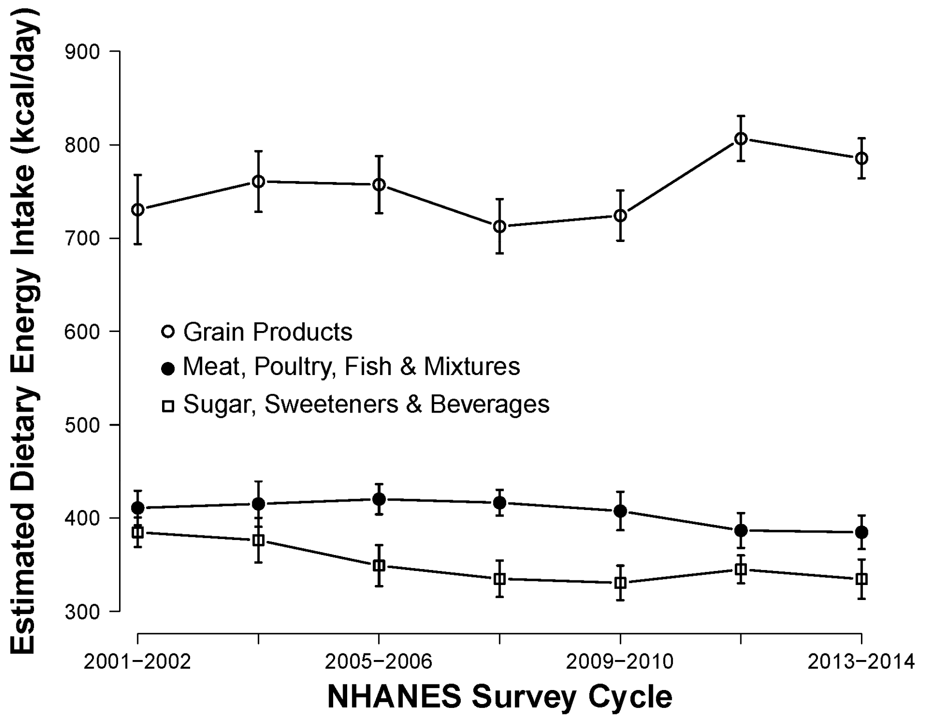

Figure S1. Estimated Total Dietary Energy Intake contributed by the top 3 USDA food groups. Error bars represent the 95% confidence interval.

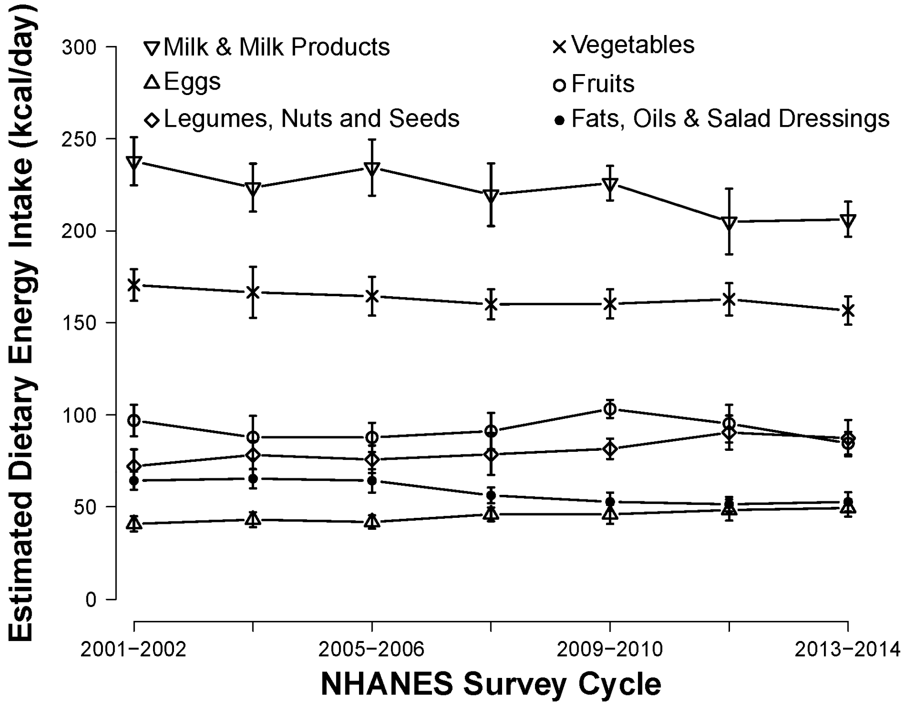

Figure S2. Estimated Total Dietary Energy Intake contributed by the bottom 6 USDA food groups. Error bars represent the 95% confidence interval.

© 2017 by the authors. Submitted for possible open access publication under the terms and conditions of the Creative Commons Attribution (CC-BY) license (http://creativecommons.org/licenses/by/4.0/).
